# New Method for the Radical Cure of Varix, and Especially of Varicocele

**Published:** 1841-05

**Authors:** 


*New method for the radical cure of Varix, and especially of Varicocele.*—By M. Ricord. After pointing out the errors of believing that varicocele affects only persons of twenty or thirty years old, and imagining it to be a common consequence of gonorrhea or epididymitis, whereas in fact it is more generally a predisposing cause of the latter disease, and instead of being produced by it, is more often cured by it; M. Ricord proceeds to describe his mode of operation.

"The hair must be shaved from the genital organs on the side to be operated on, and the veins must be dilated by making the patient walk about a little, or by enveloping the scrotum for a few hours in hot poultices, or by fomentations. This being done (though if the swelling is at all times considerable these precautions are unnecessary) the vas deferens must be separated from the mass of veins, and the latter being taken up with a fold of the scrotum, a flat lance-shaped needle, armed with a double-looped thread must be passed beneath them. When the needle has been passed completely through the skin from one side to the other, the veins are to be let go, the skin alone being now held up, and then a second needle, similarly armed, must be passed through over the veins, entering at the same hole by which the first needle was thrust out, and passing out at the same hole by which the first entered. The bundle of veins is thus fixed between two double threads, of which one passes over and the other beneath it. The ends of each double thread on each side are then to be passed into the loop of the other, and now, by drawing these ends in opposite directions, the vessels are tied beneath the skin. By this kind of ligatures the vessels may either be suddenly constricted or be tied gradually in a manner something like that adopted by M. Breschet, or most conveniently by a properly adopted *serre-nœud* after the fashion of a tourniquet.

"It is usually from the tenth to the twentieth day that the vessels are divided by this means, and their division may be easily recognized by the freedom with which the ligatures may be drawn from one side to the other, without being, as they were before, retained by the parts which they inclosed. It sometimes happens, that at the instant of the first constriction the patient suddenly feels rather an acute pain in the course of the spermatic cord; it is usually less severe than in the other operations for the same purpose; and though it often occurs at the successive constrictions, yet it has never been long continued, nor given rise to any accident. It is sufficient to keep the scrotum raised, to employ some anodyne frictions on the inguinal canal or the lumbar region, or to apply some emollient poultices, to effect its removal. Sometimes a slight œdema of the scrotum supervenes, and I have twice observed rather a considerable serous effusion in the tunica vaginalis. In one patient, also, who went out of the hospital and in a few days after the operation exposed himself to great fatigue, a slight abscess formed in the cellular tissue; but with these exceptions there has been no important accident.

"It must be clearly understood, that if the patient is strong and plethoric, he is to be bled from the arm directly after the operation; that the horizontal position must be maintained till the vessels are cut through; and that the bowels must be kept open.

"Twelve patients have now been operated on in this manner at the Venereal Hospital, and in all the most complete and satisfactory result has been obtained. The three last of them were presented at the Academy of Medicine; two completely cured, and the third, who was operated on only two days previously, still wearing the ligature and the *serre-nœud.*

"I have employed the same method for varices of the legs. I have already operated on nine patients, some having simple varicose swellings, and others varicose ulcers. In some a single ligature was sufficient, in others as many as four were applied. In none have there occurred any symptoms of phlebitis; the varicose veins have been obliterated and the ulcers speedily cicatrised; and in one of the patients whom I saw six months after the operation, there was no relapse. Still, however, I do not think that this method is likely to be so successful in all cases of varices of the lower extremities as in those of varicocele."—*Bulletin Generale de Therapeutique. Mars,* 1840. *Maryland Med. and Surg. Jour. Vol.* 1. *No.* 4.

